# EPR Spectroscopic Examination of Different Types of Paramagnetic Centers in the Blood in the Course of Burn Healing

**DOI:** 10.1155/2019/7506274

**Published:** 2019-06-19

**Authors:** Katarzyna Komosinska-Vassev, Paweł Olczyk, Janusz Kasperczyk, Barbara Pilawa, Ryszard Krzyminiewski, Bernadeta Dobosz, Paweł Ramos, Jerzy Stojko, Mateusz Stojko, Diana Ivanova, Krystyna Olczyk

**Affiliations:** ^1^Department of Clinical Chemistry and Laboratory Diagnostics, School of Pharmacy and Division of Laboratory Medicine in Sosnowiec, Medical University of Silesia in Katowice, Sosnowiec, Poland; ^2^Department of Community Pharmacy, School of Pharmacy and Division of Laboratory Medicine in Sosnowiec, Medical University of Silesia in Katowice, Sosnowiec, Poland; ^3^Centre of Polymer and Carbon Materials, Polish Academy of Sciences, Zabrze, Poland; ^4^Chair and Department of Biopharmacy, School of Pharmacy with the Division of Laboratory Medicine in Sosnowiec, Medical University of Silesia in Katowice, Poland; ^5^Department of Biophysics, School of Pharmacy with the Division of Laboratory Medicine in Sosnowiec, Medical University of Silesia in Katowice, Sosnowiec, Poland; ^6^Medical Physics Division, Faculty of Physics, Adam Mickiewicz University, Poznan, Poland; ^7^Center of Experimental Medicine, Medics 4, Faculty of Medicine in Katowice, Medical University of Silesia in Katowice, Katowice, Poland; ^8^Department of Biochemistry, Molecular Medicine and Nutrigenomics, The Faculty of Pharmacy, Medical University of Varna, Varna, Bulgaria

## Abstract

The multicomponent electron paramagnetic resonance spectra of the blood during healing of skin burned wounds treated with a new generation biodegradable dressings containing poly(lactide-co-glycolide) were analysed. The evolution of different types of paramagnetic centers in the blood with time of healing was determined. The EPR spectra of the blood samples at 230 K temperature were measured at 1, 10, and 21 days after burning of the pig skin. The EPR lines of the following paramagnetic centers: the high-spin Fe^3+^ in methemoglobin (line I), high-spin Fe^3+^ in transferrin (line II), and Cu^2+^ in ceruloplasmin and free radicals (line III) were observed in the X-band (9.3 GHz) spectra of the blood. The multicomponent structure of the EPR spectra of the tested blood samples depended on the time of the healing of the burned wounds. The amount of the high-spin Fe^3+^ in methemoglobin (line I) in the blood decreased after 21 days of the healing of the burned wounds. The amount of the high-spin Fe^3+^ in transferrin (line II) slightly increased after 21 days of therapy with the basis. The amount of Cu^2+^ in ceruloplasmin and free radicals (line III) in the blood was very high after 10 days of therapy. At the first day of the healing of the burned wounds, the highest amount of the high-spin Fe^3+^ in methemoglobin (line I), the relatively lower amounts of the high-spin Fe^3+^ in transferrin (line II), and Cu^2+^ in ceruloplasmin and free radicals (line III) existed in the blood. In the medium phase (after 10 days) of the healing of the burned wounds, the extremely higher amounts of Cu^2+^ in ceruloplasmin and free radicals (line III) appeared in the blood. In the last phase (after 21 days), only the low differences between the amounts of the high-spin Fe^3+^ in methemoglobin (line I), the high-spin Fe^3+^ in transferrin (line II), and Cu^2+^ in ceruloplasmin and free radicals (line III) were observed. The present study may serve as a starting point for the development of a new technique for monitoring molecular complexes containing iron Fe^3+^ (methemoglobin, transferrin) or copper Cu^2+^ ions (ceruloplasmin) and free radicals in the blood during wound healing.

## 1. Introduction

Thermal burns represent one of the most challenging types of trauma. Injuries range from mild reddening of the skin to severe tissue damage with additional systemic complications. The pathophysiology of thermal injury is related to the initial distribution of heat within the skin. Thermal injury is progressive and results in a spectrum of local and systemic homeostatic derangements that contribute to burn shock [1, 2]. The local response is related to cells and tissue damage caused by the thermal energy itself or by action of inflammatory mediators triggered by the initial thermal insult [3]. Oxygen free radicals, mainly from activated neutrophils, are contributory to local tissue damage following thermal injury [4, 5]. Excessive production of free radicals or proteinases can lead to the damage of skin cells and endothelial cells not affected initially by the burning insult. As a result, increased capillary permeability occurs [6]. Electrolytes and plasma move from the circulation into the interstitial spaces, and the consequences of this are hypovolaemia, decreased tissue perfusion, and oxygenation. Locally, the burn wound tends to extend in the acute phase of the injury and then to microvascular changes, profound activation of white cells and platelets, and the excessive formation of interstitial tissue oedema in the burned area and its vicinity [1].

The release of cytokines and noncytokine inflammatory mediators, as well as the excessive formation of reactive oxygen species (ROS) at the site of injury, has a systemic effect inducing hyperdynamic and hypermetabolic state that can lead to severe progressive distant organ failure [7, 8]. Moreover, oxygen radicals could contribute to secondary tissue impairment acting as harmful agents to proteins, DNA, and RNA and causing deteriorative oxidative damage. In the early stages of the systemic inflammatory process in the course of burn healing, radicals exert their actions via the activation of nuclear factors such as NF*κ*B or AP-1 and promote transcription and translation of numerous inflammatory cytokines [1, 9, 10]. The influence of free radicals in the wound healing process is discussed in a large number of paper, but none of them are related with the measurement of free radicals directly in the blood [11, 12]. Our earlier study using electron paramagnetic resonance (EPR) and the numerical procedures of the spectral line shape analysis allowed for the identification of different types of free radicals in the skin of burned wounds during the healing process [13]. Free radicals may modulate the ECM component metabolism, thus contributing to controlling the healing process at a cellular level [14]. However, the precise role of radicals in burn wound repair is still little explored.

On the other hand, except for the increased formation of free radicals, the local and systemic inflammatory response to thermal injury involves the increased hepatic synthesis of a number of plasma proteins, which can markedly facilitate wound healing. Plasma proteins can support tissue repair by metabolic and functional activity. Among them, ceruloplasmin (Cp), transferrin (Tf), and hemoglobin (Hb) play an important role in EPR studies. Ceruloplasmin, the primary Cu-transporting protein, is an acute phase reactant synthesized in the liver and a multifunctional enzyme, which takes part in the wound healing process [15]. Copper is known to be a critical component of lysyl oxidase (LOX), the enzyme required for the cross-linking and maturation of collagen. Through its promotion of vascular endothelial growth factor, copper may accelerate wound healing by stimulating angiogenesis [16, 17]. Another EPR investigations of paramagnetic centers in the whole blood during the healing process after burn traumatic injury included the molecular complexes containing Fe^3+^ such as transferrin and methemoglobin. Fe was postulated as beneficial in wound healing, especially due to its status as a cofactor for both lysyl and prolyl hydroxylase in collagen synthesis. In wound healing, iron deficiency results in decreased collagen synthesis in the proliferative phase as well as in impaired T cell and phagocyte function in the inflammatory phase. Finally, as a critical component of hemoglobin, iron is beneficial in wound healing providing oxygen transport to proliferating tissues [18, 19]. Whereas iron, copper, and free radicals represent highlight factors that can regulate wound healing at cellular and molecular levels, in this work, the EPR signals from high-spin Fe^3+^ ions in transferrin (*g* = 4.14) and methemoglobin (*g* = 5.89) and Cu^2+^ ions in ceruloplasmin and free radicals (*g* = 2.05) were analysed in the blood during healing of the burned wounds.

## 2. Experimental

### 2.1. Samples

Two 16-week-old domestic pigs were implemented for the evaluation of wound repair because of the many similarities between porcine skin and human skin. The contact thermal injuries were inflicted according to the methods of Hoekstra et al. [20]. The wounds were then covered with a new generation biodegradable dressings. Nonwoven wound dressings were obtained by an electrospinning method using poly(lactide-co-glycolide) containing 85 mole-% of lactidyl and 15 mole-% of glycolidyl comonomeric units (PLGA 85 : 15) [21]. 1,1,1,3,3,3-Hexafluoro-2-propanol as a solvent and 6 wt-% concentration of the polymer in solution were used. The electrospinning process consists in producing the polymer fibers in the electric field between the spinning nozzle to which the positive electric potential has been applied and the collector with negative electric potential [22]. The potential difference was adjusted to 27 kV. The distance between the electrodes has been set to 15 cm. To obtain the nonwoven mat, 22 ml of solution was dosed at a rate of 1.5 ml/h [23].

Biopsies, in three replications, were taken from the wound bed on postburn days 1, 10, and 21. The wounds left by the biopsy were covered with collagen dressing. Experimental animals were housed according to the Good Laboratory Practice Standards.

Venous blood was collected from young animals using the marginal ear vein method. The pig was restrained, and the ear was cleaned with three alternating scrubs of 70% alcohol and betadine. Blood collection was performed on the vein puncture at the base of the lateral surface of the ear. When the vein was punctured, the emerging blood was collected directly by capillary action into EDTA vacutainer tubes. After collection, the blood was stored at low temperature (-70°C).

The experimental protocol was accepted by the Ethics Committee of the Medical University of Silesia in Katowice, Poland (LKE-111/2014).

### 2.2. EPR Measurements

For the blood samples, electron paramagnetic resonance (EPR) spectra were measured. The microwaves with a frequency of 9.3 GHz from the X-band were used. The EPR lines of paramagnetic centers in the tested blood samples were recorded by the EPR spectrometer produced by Bruker (USA) at low temperature equal to 230 K. The cryogenic system to low-temperature measurements of Bruker (USA) was used. The spectra were measured with a magnetic modulation frequency of 100 kHz as the first-derivative curves. To avoid microwave saturation of the lines, all the EPR spectra were obtained with the low microwave power of 7.9 mW. The microwave power was measured by the recorder of Bruker (USA).

The following parameters of EPR spectra of the blood samples were analysed: *g* factors and amplitudes (*A*). *g* values will be calculated from a resonance condition according to the following formula [24–26]:

(1) $$\begin{array}{c} g=\frac{hv}{\mu_{B}B_{r}}, \end{array}$$

where *h* is the Planck constant, *v* is the microwave frequency, *μ*_*B*_ is the Bohr magneton, and *B*_*r*_ is the resonance magnetic field.

The microwave frequency (*ν*) was directly measured by the recorder in the Bruker (USA) spectrometer. The *B*_*r*_ values was determined from the electron paramagnetic resonance lines.

## 3. Results and Discussion

Wound healing, physiologic response to tissue injury, proceeds as a complex pathway of biochemical reactions and cellular events, secreted growth factors, cytokines, and noncytokine mediators of inflammation. Throughout four distinct but overlapping phases, free radicals and some proteins and trace elements, their care has been shown to play an important role in each wounding stage, including inflammation, proliferation, angiogenesis, and maturation. Oxygen free radicals are contributory agents to local tissue damage following burn injury. Moreover, thermal injury initiates systemic inflammatory reactions producing oxygen radicals [1–3]. The release of cytokines and other inflammatory mediators at the site of injury has a systemic effect. In contrast to the inflammation, which is initiated almost immediately after the burn injury, the systemic response progresses with time, usually peaking 5 to 7 days after the burn injury. The local and systemic response to thermal injury involves the increased hepatic synthesis of plasma proteins, including among others ceruloplasmin (Cp) and transferrin (Tf). They can support tissue repair by metabolic and functional activity, including the transport of trace metal cofactors such as copper and iron [11, 12, 14]. In this work, the EPR signals from high-spin Fe^3+^ ions in transferrin (*g* = 4.14) and methemoglobin (*g* = 5.89) as well as Cu^2+^ ions in ceruloplasmin and free radicals (*g* = 2.05) were recorded in the blood during the burn wound healing.

**Figure 1:**
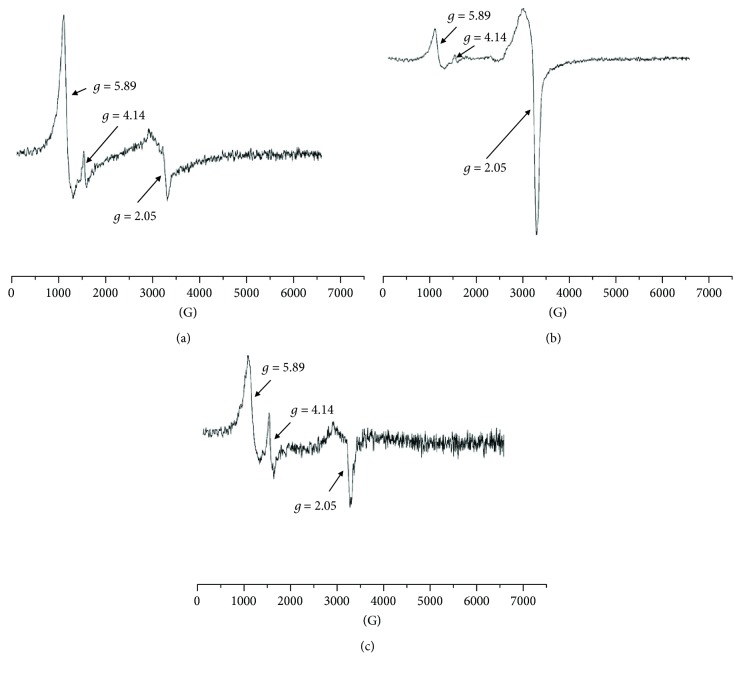
The first derivative EPR spectrum of paramagnetic centers: the high-spin Fe^3+^ in methemoglobin (line I), high-spin Fe^3+^ in transferrin (line II), and Cu^2+^ in ceruloplasmin and free radicals (line III), in the blood for skin burned wounds treated with new generation biodegradable dressings containing poly(lactide-co-glycolide), at (a) 1 day, (b) 10 days, and (c) 21 days of therapy.

**Figure 2:**
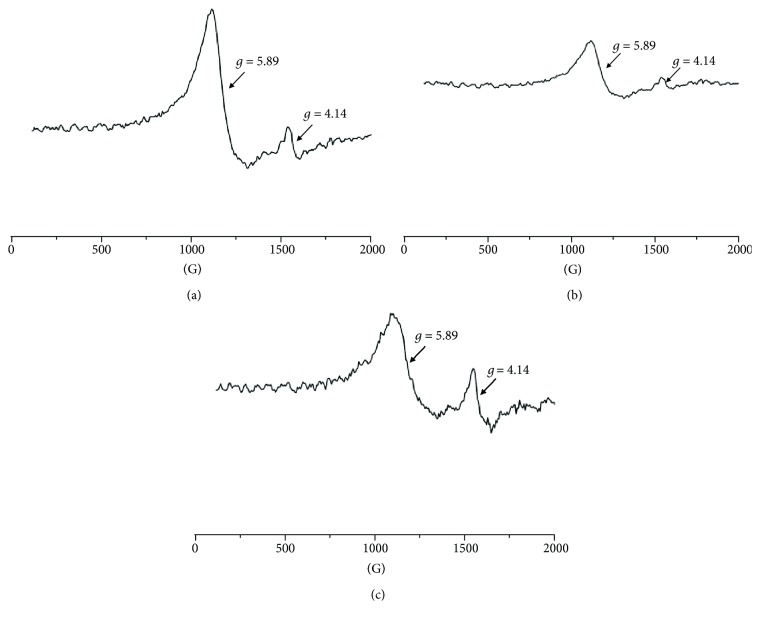
The EPR lines of the high-spin Fe^3+^ in methemoglobin (line I) and high-spin Fe^3+^ in transferrin (line II), in the blood for skin burned wounds treated with new generation biodegradable dressings containing poly(lactide-co-glycolide), at (a) 1 day, (b) 10 days, and (c) 21 days of therapy.

Several lines were observed in the EPR spectra of the blood samples obtained from the experimental animals during different phases of burn healing covered with new generation biodegradable dressings containing poly(lactide-co-glycolide) (PLGA). The shape of these spectra clearly indicated their multicomponent character. The component lines appeared at different magnetic fields. The EPR spectra of the blood for skin burned wounds treated with the new generation PLGA-containing dressing for 1 day, 10 days, and 21 days of therapy are shown in Figures 1(a)–1(c), respectively. The EPR lines of the high-spin Fe^3+^ in methemoglobin (line I), high-spin Fe^3+^ in transferrin (line II), and Cu^2+^ in ceruloplasmin and free radicals (line III) were visible in the complex spectra. The measured EPR spectra of the blood were superposition of these three signals. The third EPR component line (line III) was unresolved, and it was the sum of EPR lines of paramagnetic Cu^2+^ ions and free radicals (*S* = 1/2). The EPR signals of Cu^2+^ and free radicals overlapped, and they give complex line III in the whole spectrum of the tested blood samples. The paramagnetic centers with the individual EPR components (lines I, II, and III) absorbed different amounts of microwaves that reflected their different fractions in the whole paramagnetic system in the tested blood. The low field signals (line I, line II) of the EPR spectra of the blood samples in the experimental measure for the bandage containing only the basis for 1 day, 10 days, and 21 days of therapy are presented in Figures 2(a)–2(c), respectively.

Taking to account that obtained EPR were recorded at *g* = 5.89, *g* = 4.14, and *g* = 2.05, they represent typical signals of paramagnetic centers in the blood: the high-spin Fe^3+^ in methemoglobin (line I), high-spin Fe^3+^ in transferrin (line II), and Cu^2+^ in ceruloplasmin and free radicals (line III), respectively [15, 19]. In this work, the performed EPR examination shows the evolution of the individual types of paramagnetic centers existing in the blood during healing of experimental burned wounds.

**Figure 3:**
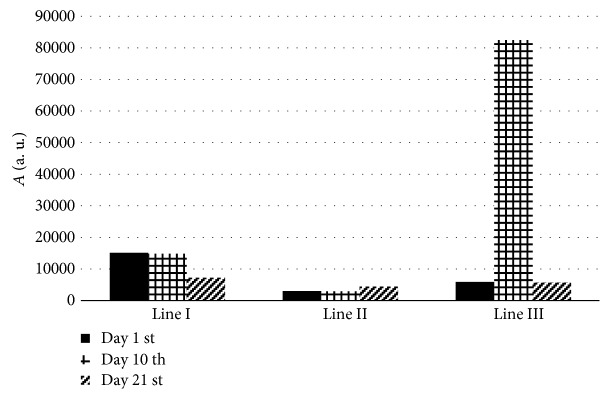
The influence of time of therapy for the amplitudes (*A*) of the high-spin Fe^3+^ in methemoglobin (I), high-spin Fe^3+^ in transferrin (II), and Cu^2+^ in ceruloplasmin and free radicals (III), in the blood for skin burned wounds treated with new generation biodegradable dressings containing poly(lactide-co-glycolide). The data obtained after 1 day, 10 days, and 21 days of therapy were compared.

Comparing the multicomponent structures of the EPR spectra of the tested blood samples presented in Figures 1 and 2, it may be seen that the amplitudes (*A*) of the individual EPR component lines in the spectral changes during the healing process. The amplitudes (*A*) of the individual lines (lines I, II, and III) changed differently with time of therapy. Such conclusion was done from the analysis of line I, line II, and line III, in the EPR spectra of the blood. Their amplitudes were compared in the diagram in Figure 3, where the amplitudes (*A*) of the individual component lines (I, II, and III) were presented for the spectral observations at the 1 day, 10 days, and 21 days of the healing of the burned wounds dressed by nanofiber dressing containing poly(lactide-co-glycolide).

The amount of the high-spin Fe^3+^ in methemoglobin responsible for the first line (line I) in the EPR spectra of blood samples remains stable up to 10 days of the experiment (Figure 3). In the late phase of the healing process, after 21 days, the amount of the high-spin Fe^3+^ in methemoglobin decreased compared to the EPR signals obtained from the blood samples collected on days 1 and 10 of the experiment (Figure 3). These relations are reflected by the considerably lower amplitudes of the EPR line (line I) after 21 days of healing than the amplitudes after 1 day and 10 days of the burning. The observed changes in the EPR signal from high-spin Fe^3+^ in methemoglobin which occurs in response to injury reflect an essential contribution of these molecules to the processes involved in wound healing. Methemoglobin is a poor oxygen carrier, which is formed *in vivo* at low levels at physiological conditions and at much higher levels in the presence of oxidative compounds [18]. Excessive presence of this protein is disadvantageous; the more so as a result of its reaction with hydrogen peroxide, free radicals are formed in the blood. Thus, the decrease in EPR from high-spin Fe^3+^ in methemoglobin observed in the final phase of the healing process may be a favorable indicator of suppression of oxidative stress conditions and proper course of the healing process.

The characteristic for the high-spin Fe^3+^ in transferrin (line II) was only the low changes of the amount of these types of paramagnetic centers in the blood during the healing of the burned wounds (Figure 3). Similar amounts of the high-spin Fe^3+^ in transferrin (line II) existed in the blood samples obtained during days 1 and 10 of the experiment. The slight increase of the amount of the high-spin Fe^3+^ in transferrin (line II) was observed after 21 days of therapy of the burned wounds with the nanofiber coverage (Figure 3). The role of transferrin in the healing process that occurs after thermal burn is complex. Tf constitutes a part of the defense system against the excess of free iron. This glycoprotein prevents iron-mediated free radical toxicity and facilitates the transport of iron into the cells. Taking into account that transferrin is the most important physiological source of iron for erythropoiesis, which is markedly disturbed after burn injury, investigation of the electron states of iron complexes in the human blood by EPR spectroscopy could give important information concerning the proper providing of oxygen to cell metabolism and nearly all wound healing processes. Oxygen helps prevent wounds from infection; increases keratinocyte differentiation, migration, and reepithelialization; and promotes wound contraction [27]. Thus, the mechanism by which iron deficiency may impair wound healing involves hypoxia. Decreased molecular oxygen in a wound results in the formation of a nonhealing ulcer or impaired healing [28]. Hypoxia can also act as a stimulus to fibroblast proliferation and angiogenesis, but proper levels of oxygen are absolutely essential for synthesis and cross-linking of collagen [27, 29]. Iron is a necessary cofactor for both lysyl and prolyl hydroxylases required for hydroxylation of proline and lysine residues in collagen fibers. Due to its status as a cofactor in collagen synthesis, Fe was postulated as a beneficial factor in wound healing. However, considering the role of iron in the Fenton reaction, which is implicated in the formation of hydroxyl radicals, recent studies suggest that iron may retard wound healing through its action as a critical factor related to persistent inflammation and increased tissue destruction induced by ROS generation [28].

In addition to the increased production of free radicals, thermal injury elicits a number of other metabolic responses, including increased synthesis of the acute phase proteins, among which the essential role in the wound healing process is played by ceruloplasmin. Thus, we analysed the EPR spectra recorded at *g* = 2.05, representing a typical signal from Cu^2+^ ions in ceruloplasmin and free radicals. The amounts of Cu^2+^ in ceruloplasmin and free radicals (line III) in the blood samples were similar on the 1^st^ and 21^st^ days of the healing of the burned wounds (Figure 3). Significantly increased blood amount of Cu^2+^ in ceruloplasmin and free radicals (line III) was noticed on the 10^th^ day of healing. It was reflected by the extremely strong increase of the amplitude of the EPR line of Cu^2+^ in ceruloplasmin and free radicals (line III) in the blood (Figure 3).

Ceruloplasmin, the primary Cu-transport protein, takes part in the wound healing process as an acute phase reactant and multifunctional enzyme, which shows aminooxidase, superoxide-dismutase, and ferrooxidase activities. Copper is known to be a critical component in lysyl oxidase (LOX), the enzyme required for the cross-linking and maturation of collagen [17, 30]. Ceruloplasmin supplies copper to the superoxide dismutase (SOD), a scavenger of oxygen free radicals, thus protecting the matrix of healing tissue against superoxide ions, generated by activated neutrophils during inflammatory phase. Both copper and ceruloplasmin play an important role in iron metabolism and transfer and hemoglobin synthesis. Ceruloplasmin can be considered a genuine link between copper metabolism and iron metabolism. It catalyzes the oxidation of iron Fe^2+^ to Fe^3+^, which can be included into transferrin [30]. A deficit in copper reduces the ferroxidase activity of ceruloplasmin (Fe^2+^ to Fe^3+^). Moreover, Cu has been recognized as a regulator of angiogenesis; however, specific targets of Cu action and detailed mechanism remain unknown [17]. A significant increase in EPR signal from Cu^2+^ ions in ceruloplasmin and from free radicals (*g* = 2.05) recorded in the blood on the 10^th^ day of the burn healing process was found in our study, indicating that copper and free radical are essential participants in the late phase of inflammation, and the early phase of proliferation and remodeling, which occur at this time.

On the other hand, due to its potential toxicity in excess, metabolism of copper should be under tight control, in order to maintain free copper concentrations at very low levels. The intracellular copper is regulated by transmembrane copper transporters such as CTR1, ATP7A, and ATP7B, as well as the copper chaperones, including CCS: chaperone for superoxide dismutase 1; Cox17: chaperone for cytochrome C oxygenase; and Atox1 antioxidant-1: chaperone for the ATPases, ATP7A, and ATP7B. It has been found by Das et al. [31] that wound injury selectively increases expression of endothelial cells' Atox1, but not other Cu chaperones. It has been found that Atox1 in the cytosol delivers Cu to Cu-dependent enzymes such as extracellular superoxide dismutase (ecSOD) or lysyl oxidase (LOX) regulating the crosslinking of collagen, which plays an important role in tissue remodeling [30, 31] Moreover, Atox-1 plays a role as a Cu-dependent transcription factor for cyclin D1 to promote cell proliferation or for cytosolic NADPH oxidase organizer p47phox to promote ROS production and inflammatory cell recruitment, both required for reparative neovascularization and wound healing tissue repair [31]. Copper also can change the structure and activity of matrix metalloproteinases (MMPs), enzymes wherein their role in the healing process is already evident in the inflammatory phase, where they participate in phagocytosis and killing bacteria and removing dead tissue fragments. MMPs are also involved in the remodeling phase, conditioning the separation of keratinocytes from basal membranes and supporting the migration of keratinocytes through the collagen network. During angiogenesis, MMPs digesting the basal membranes determine the migration of endothelial cells and release growth factors, sequestered in the ECM [27, 32]. On the other hand, copper overload shifts balance between the reduced and oxidized forms of glutathione (GSH and GSSG, respectively) making cellular environment more oxidizing [33, 34].

The comparative analysis of the EPR spectra resulting from different types of paramagnetic centers in the blood was useful to describe the evolution of paramagnetic centers during the healing process in the example of the burned wounds. Information about paramagnetic centers as the units with unpaired electrons was helpful to describe the healing process of the burned wounds.

## 4. Conclusions

The X-band (9.3 GHz) electron paramagnetic resonance (EPR) examination of the paramagnetic centers in the blood obtained from the pig during healing the burned wounds dressed with a new generation biodegradable dressings containing poly(lactide-co-glycolide) indicated the following:

1. The multicomponent EPR spectra as the superposition of the lines of the high-spin Fe^3+^ in methemoglobin (line I), high-spin Fe^3+^ in transferrin (line II), and Cu^2+^ in ceruloplasmin and free radicals (line III) were characteristic for the analysed blood samples and were dependent on the time of the healing of the burned wounds
2. The amount of the high-spin Fe^3+^ in methemoglobin (line I) in the blood did not change up to 10 days, and it decreased after 21 days of the healing of the burned wounds
3. The amount of the high-spin Fe^3+^ in transferrin, which was responsible for the second EPR component (line II), slightly increased on the 21st day of therapy with the use of biodegradable dressing
4. The amounts of Cu^2+^ in ceruloplasmin and free radicals (line III) in the blood on the 1st and 21st postburn days of the healing of the burned wounds were similar. The amplitude of the EPR line (line III) and the amount of Cu^2+^ in ceruloplasmin and free radicals (line III) in the blood were strongly the highest on the 10^th^ postburn day
5. At the first day of the healing of the burned wounds, the highest amount of paramagnetic centers in the form of the high-spin Fe^3+^ in methemoglobin (line I) existed in the blood. The relatively lower amounts of the high-spin Fe^3+^ in transferrin (line II) and Cu^2+^ in ceruloplasmin and free radicals (line III) were observed
6. In the medium phase of the healing (10 days after burn), extremely high amounts of Cu^2+^ in ceruloplasmin and free radicals (line III) existed in the blood. The lowest amount of the high-spin Fe^3+^ in transferrin (line II) compared to the Cu^2+^ in ceruloplasmin and free radicals (line III) and the high-spin Fe^3+^ in methemoglobin (line I) was present in the blood
7. In the last phase of the repairing process (21 days after burn), only the low differences between the amounts of paramagnetic centers in the form of the high-spin Fe^3+^ in methemoglobin (line I), the high-spin Fe^3+^ in transferrin (line II), and Cu^2+^ in ceruloplasmin and free radicals (line III) were observed
8. The results of the research may contribute to a more complete assessment of biochemical changes taking place in the process of repairing tissue damage after burn insult, in the scope of mechanisms regulating the metabolism of iron and copper ion complexes as well as free radicals in the blood

## Data Availability

The WINEPR data used to support the findings of this study have been deposited in the computer that supports an EPR spectrometer produced by Bruker (USA) repository, Medical Physics Division, Faculty of Physics, Adam Mickiewicz University, Poznan, Poland. The contact person is Professor Ryszard Krzyminiewski (rku@amu.edu.pl 2). The electrospinning method data used to obtain samples of biodegradable, nonwoven dressings are patent-protected and so cannot be made freely available. Request for access to these data should be made to Professor Janusz Kasperczyk (jkasperczyk@cmpw-pan.edu.pl) or Mateusz Stojko (mstojko@cmpw-pan.edu.pl).

## Supplementary Materials

Supplementary Materials Description of the supplementary material (graphical abstract). Nonwoven wound dressings were obtained by an electrospinning method using poly(lactide-co-glycolide) containing 85 mole-% of lactidyl and 15 mole-% of glycolidyl comonomeric units (PLGA 85 : 15). Two 16-week-old domestic pigs were implemented for the evaluation of the wound repair process. The contact thermal injuries were inflicted according to the Hoekstra model. The wounds were covered with electrospun dressings. Venous blood samples were collected on the postburn days 10, 15, and 21. Electron paramagnetic resonance (EPR) spectra of high-spin Fe^3+^ in methemoglobin (I), high-spin Fe^3+^ in transferrin (II), Cu^2+^ in ceruloplasmin, and free radicals (III) were measured. The obtained results may contribute to a more complete assessment of biochemical changes taking place in the process of repairing tissue damage after burn infliction, in the scope of mechanisms regulating the metabolism of iron and copper ion complexes as well as free radicals in the blood.
