# Supplementary figures and images for: EPR Spectroscopic Examination of Different Types of Paramagnetic Centers in the Blood in the Course of Burn Healing
Source: Oxid Med Cell Longev. 2019 Jun 19;2019:7506274. doi: 10.1155/2019/7506274 (PMC6607714; doi:10.1155/2019/7506274)

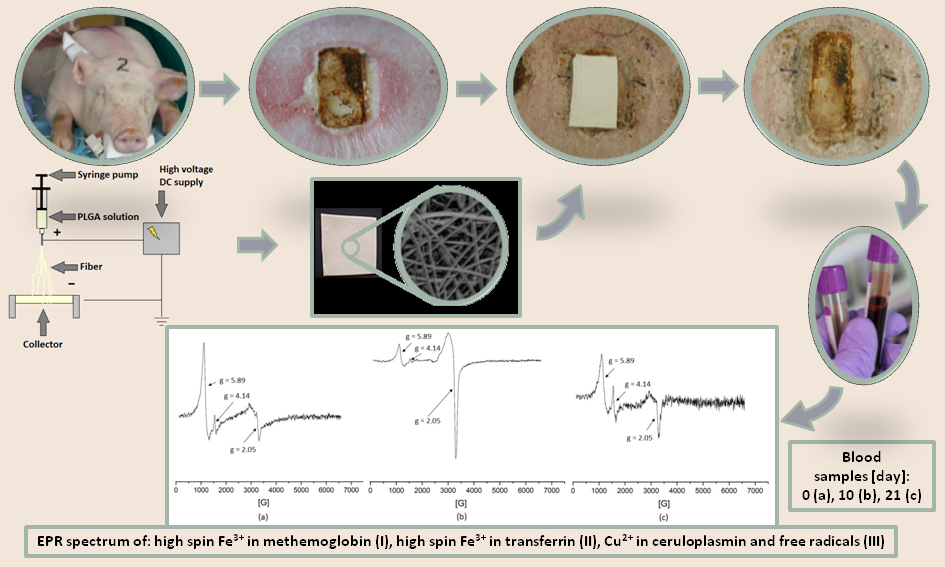

Supplement: Supplementary Materials — Description of the supplementary material (graphical abstract). Nonwoven wound dressings were obtained by an electrospinning method using poly(lactide-co-glycolide) containing 85 mole-% of lactidyl and 15 mole-% of glycolidyl comonomeric units (PLGA 85 : 15). Two 16-week-old domestic pigs were implemented for the evaluation of the wound repair process. The contact thermal injuries were inflicted according to the Hoekstra model. The wounds were covered with electrospun dressings. Venous blood samples were collected on the postburn days 10, 15, and 21. Electron paramagnetic resonance (EPR) spectra of high-spin Fe3+ in methemoglobin (I), high-spin Fe3+ in transferrin (II), Cu2+ in ceruloplasmin, and free radicals (III) were measured. The obtained results may contribute to a more complete assessment of biochemical changes taking place in the process of repairing tissue damage after burn infliction, in the scope of mechanisms regulating the metabolism of iron and copper ion complexes as well as free radicals in the blood. [file 7506274.f1.png]
